# Optimising the diagnosis and referral of achondroplasia in Europe: European Achondroplasia Forum best practice recommendations

**DOI:** 10.1186/s13023-022-02442-2

**Published:** 2022-07-27

**Authors:** Valerie Cormier-Daire, Moeenaldeen AlSayed, Inês Alves, Joana Bengoa, Tawfeg Ben-Omran, Silvio Boero, Svein Fredwall, Catherine Garel, Encarna Guillen-Navarro, Melita Irving, Christian Lampe, Mohamad Maghnie, Geert Mortier, Sérgio B. Sousa, Klaus Mohnike

**Affiliations:** 1 Department of Clinical Genetics, Centre of Reference for Constitutional Bone Diseases (MOC), INSERM UMR 1163, Imagine Institute, Necker-Enfants Malades Hospital, Paris Centre University, Paris, France; 2 Department of Medical Genetics, King Faisal Specialist Hospital and Research Center, Riyadh, Kingdom of Saudi Arabia; 3 Faculty of Medicine, Alfaisal University, Riyadh, Kingdom of Saudi Arabia; 4 ANDO Portugal, Evora, Portugal; 5 Hôpital Necker-Enfants Malades, Paris, France; 6 Division of Genetics and Genomic Medicine, Sidra Medicine & Hamad Medical Corporation, Doha, Qatar; 7 Pediatric Orthopaedic and Traumatology Unit, Istituto Giannina Gaslini, Genoa, Italy; 8 TRS National Resource Centre for Rare Disorders, Sunnaas Rehabilitation Hospital, Nesodden, Norway; 9 Department of Radiology, Armand-Trousseau Hospital, Paris, France; 10 Medical Genetics Section, Department of Pediatrics, Virgen de la Arrixaca University Hospital, IMIB-Arrixaca, University of Murcia-UMU, Murcia, Spain; 11 Department of Clinical Genetics, Guy’s and St Thomas’ NHS Foundation Trust, London, UK; 12 Clinic of Neuropediatrics, Epileptology and Social Pediatrics, University Hospital Giessen and Marburg, Giessen, Germany; 13 Department of Pediatrics, IRCCS Istituto Giannina Gaslini, 16147 Genoa, Italy; 14 Department of Medical Genetics, and Centre for Rare Diseases, UZ Leuven, Leuven, Belgium; 15 Medical Genetics Unit, Hospital Pediátrico, Centro Hospitalar e Universitário de Coimbra, Coimbra, Portugal; 16 Portugal AND University Clinic of Genetics, Faculty of Medicine, Universidade de Coimbra, Coimbra, Portugal; 17 Central German Competence Network for Rare Diseases (ZSE), Universitätskinderklinik, Otto-von-Guericke Universität, Magdeburg, Germany; 18 CIBERER-ISCIII, Madrid, Spain; 19 Department of Neuroscience, Rehabilitation, Ophthalmology, Genetics, Maternal and Child Health, University of Genova, 16147 Genoa, Italy

**Keywords:** Achondroplasia, Diagnosis, Referral, European Achondroplasia Forum, Guiding Principles

## Abstract

**Background:**

Achondroplasia is the most common form of skeletal dysplasia, with serious comorbidities and complications that may occur from early infancy to adulthood, requiring lifelong management from a multidisciplinary team expert in the condition The European Achondroplasia Forum guiding principles of management highlight the importance of accurate diagnosis and timely referral to a centre specialised in the management of achondroplasia to fully support individuals with achondroplasia and their families, and to appropriately plan management. The European Achondroplasia Forum undertook an exploratory audit of its Steering Committee to ascertain the current situation in Europe and to understand the potential barriers to timely diagnosis and referral.

**Results:**

Diagnosis of achondroplasia was primarily confirmed prenatally (66.6%), at Day 0 (12.8%) or within one month after birth (12.8%). For suspected and confirmed cases of achondroplasia, a greater proportion were identified earlier in the prenatal period (87.1%) with fewer diagnoses at Day 0 (5.1%) or within the first month of life (2.6%). Referral to a specialist centre took place after birth (86.6%), predominantly within the first month, although there was a wide variety in the timepoint of referral between countries and in the time lapsed between suspicion or confirmed diagnosis of achondroplasia and referral to a specialist centre.

**Conclusions:**

The European Achondroplasia Forum guiding principles of management recommend diagnosis of achondroplasia as early as possible. If concerns are raised at routine ultrasound, second line investigation should be implemented so that the diagnosis can be reached as soon as possible for ongoing management. Clinical and radiological examination supported by molecular testing is the most effective way to confirm diagnosis of achondroplasia after birth. Referral to a centre specialised in achondroplasia care should be made as soon as possible on suspicion or confirmation of diagnosis. In countries or regions where there are no official skeletal dysplasia reference or specialist centres, priority should be given to their creation or recognition, together with incentives to improve the structure of the existing multidisciplinary team managing achondroplasia. The length of delay between diagnosis of achondroplasia and referral to a specialist centre warrants further research.

## Background

Achondroplasia is the most common form of skeletal dysplasia, with an estimated prevalence of 3.7–4.6 per 100,000 births [1, 2]. It is characterised by disproportionate short stature, macrocephaly, frontal bossing, trident-shaped hands, near normal trunk length and normal cognition [3–5]. Achondroplasia is an autosomal dominant disorder, caused by a recurrent pathogenic variant in the fibroblast growth factor receptor 3 (*FGFR3*) gene [4, 6]. There is no gender or ethnic disposition, and approximately 75–80% of individuals with achondroplasia are born to average-stature parents, indicating a new mutation in the *FGFR3* gene in these individuals [5–7]. People with achondroplasia have a normal, or near normal life expectancy [5] and require lifelong management by an experienced multidisciplinary team (MDT) [8]. Co-morbidities throughout the lifetime of an individual with achondroplasia may include spinal stenosis, thoracolumbar kyphosis, sleep apnoea, obesity, and pain [9–14], all of which can impact significantly on quality of life and mental health [15–18]. Serious complications may occur in early infancy, including craniocervical junction compression, otitis media, craniofacial issues, hydrocephalus, restrictive breathing problems and central apnoea [5, 9, 10, 19], underlining the need for specialist management.

The time to reach a diagnosis and referral to a specialist centre can be a challenging in rare diseases, resulting in misdiagnoses, unnecessary hospital visits and procedures [20]. There may also be a psychological impact on the individual and their families [20]. A EURORDIS survey in 2007 on eight relatively common rare diseases (Crohn’s disease, cystic fibrosis, Duchenne muscular dystrophy, Ehlers Danlos syndrome, Marfan syndrome, Prader-Willi syndrome, tuberous sclerosis, and Fragile X syndrome) indicated that lack of referral was often cited as a reason for inaccessibility of healthcare services [20]. It is vital to understand the process of diagnosis and referral in rare diseases to improve overall care.

### Prenatal indicators of achondroplasia

There are several indicators that can prompt suspicion of achondroplasia *in utero*, either at routine ultrasound screening or on further investigation, however, the timing of routine ultrasound can preclude the identification of achondroplasia *in utero*. Limb length is often preserved until after the time of the fetal anomaly scan undertaken at approximately 20–25 weeks gestation [21]. With shortening of the femora not usually apparent until 25 weeks’ gestation [21], unless there is a routine scan or other indication for further imaging after the fetal anomaly scan, the condition may not be identified prenatally. If reduced length of the femur or other long bones (measuring <3^rd^ centile) is observed on routine ultrasound screening [21], further ultrasonography and investigation for other diagnostic features is recommended [5]. Head circumference >95th centile may also be an indicator of achondroplasia [21]. Second-line ultrasound investigation may identify a widening and rounded appearance of the metaphysis-diaphysis angle >90°, which is present from 20 to 24 weeks’ gestation [22] and is a constant finding in foetuses with achondroplasia [23]. Other indicators to prompt suspicion of achondroplasia on ultrasound evaluation include the echogenic “collar hoop” sign described by Boulet et al. [24] evidence of frontal bossing, depressed nasal bridge, short fingers (trident hand) [21], and mild platyspondyly. In addition, *FGFR3*-associated medial temporal lobe dysplasia may be seen, although this is not common. [25]

In the event that ultrasound markers do not readily indicate the specific underlying diagnosis, computed tomography (CT) imaging may be beneficial to identify differentiating features of other skeletal dysplasia conditions, to expedite diagnosis in advance of molecular test results, and to provide additional anatomical clues to optimise diagnostic accuracy where local expertise is lacking. It is possible to assess the appearance of the pelvic bones as well as narrowing of the spinal canal on CT [26]; neither is well-depicted on ultrasound. Prenatal CT is not commonly used in all European countries for diagnostic purposes, and the levels of radiation to the fetus have historically been of concern. However, the mean radiation dose is relatively low and is maintained at ≅4.8 mSv and the average computed tomography dose index is ≅5.9 mGY. CT is routinely performed to confirm a diagnosis in France and Belgium, and at some centres in the USA [27, 28]. CT can be a useful additional option in diagnosing achondroplasia. The place of fetal MRI in the diagnostic pathway is, as yet, unclear.

### Postnatal indicators of achondroplasia

There are well defined clinical indicators of achondroplasia that are evident soon after birth [9], including reduced birth length, macrocephaly, frontal bossing and depressed nasal bridge, short fingers with trident configuration of the hands, small chest and relative hypotonia [5, 9]. Characteristic radiological features include shortening of the long bones, abnormal pelvis with squared iliac wings and narrow sacrosciatic notches, flat acetabular roof, oval-shaped lucent appearance of the proximal femur, mild flattening and dorsal scalloping of the vertebral bodies, and a decrease of the interpedicular distance from the upper to the lower lumbar spine [5]. The diagnosis of achondroplasia can be made based on clinical and radiographic assessment alone [9, 29], although this may be problematic in the instance of preterm birth [30]. As with ultrasound diagnosis, radiological diagnosis may depend on the expertise of the examiner.

### Confirming diagnosis

Since the identification of the *FGFR3* gene as the causal gene for achondroplasia [6], the diagnosis can be easily confirmed by genetic analysis, either postnatally or prenatally [19]. Timely confirmation of diagnosis is important to equip parents with accurate information for decision making.

### Referral to a specialist achondroplasia centre

Recent guidelines recommend that referral to a physician experienced in achondroplasia should be made as soon as possible when the diagnosis is made or suspected, either pre- or postnatally, to enable genetic counselling and discussion of prognosis and management. [8, 19, 31]

Genetic counselling for the family at the time of diagnosis is important to aid in decision making, whether the parents are affected by achondroplasia, or if there is a *de novo* mutation [10]. Physicians not experienced in achondroplasia may lack the knowledge to effectively counsel families [32], so referral to, or communication with, a specialist centre is advised for guidance on care [8]. Savarirayan et al. recommend that in cases with a confirmed prenatal diagnosis of a skeletal dysplasia, the pregnant mother is referred to a centre where a high-risk maternal-fetal unit is available [31]. There are considerations for delivery of a child with achondroplasia, such as avoidance of instrumentation due to increased risk of intracranial and cervical spine complications, and newborns may require immediate medical management. [31]

There are clinical considerations for newborns with achondroplasia including, among others, spine care to mitigate against persistence of thoracolumbar kyphosis after unsupported sitting [19, 33], detailed sleep study to assess for sleep disordered breathing [5], and evaluation of the foramen magnum and craniocervical junction, which may require surgical intervention in early infancy if compression is apparent [9, 34]. The American Academy of Pediatrics Recommendations state that the following assessments should be carried out within the first month after birth: neurologic evaluation with neuroimaging and assessment by an experienced neurosurgeon (if necessary), sleep study, and audiology assessment [5]. In addition, anticipatory guidance indicates considerations for early infant handling, including appropriate use of car seats and baby carriers, breast- and bottle-feeding positioning, and avoidance of unsupported sitting. [5, 9, 19, 33, 35]

Psychological and peer support for families on receiving a diagnosis of achondroplasia is recommended, in addition to resources to aid informed decision making for the management of the pregnancy and initial clinical management of the infant [5]. Connection to a patient advocacy group at the point of diagnosis can help to support the parents with timely information, assistance understanding the implications of the diagnosis and peer and psychological support. [32, 36]

The European Achondroplasia Forum (EAF) have recently published six principles for the management of achondroplasia (Table 1) [8]. These provide a basis for the optimal care of individuals with achondroplasia, and a framework within which aspects of achondroplasia care can be assessed in greater detail. With the practical considerations for delivery of a child with achondroplasia, and those for newborns, coupled with the potential for serious and life-threatening complications that may occur in infancy and early childhood, timely diagnosis and referral to a specialist in the management of achondroplasia are vital. The EAF guiding principles state: *When a diagnosis of achondroplasia is made or suspected, either in utero or after birth, the family should be referred as soon as possible to a physician experienced in achondroplasia to discuss the prognosis and management of the condition*. This relies on timely and accurate diagnosis, however there is no published literature on the timing of diagnosis or referral, or evidence of diagnostic delay. To gain a greater understanding of the process of diagnosis and referral, and whether it is indeed timely, the EAF undertook an audit of their Steering Committee to establish the prenatal diagnostic pathway in Europe, and to combine their expert opinion to establish recommendations for best practice pathways for diagnosis and referral.

**Table 1:** The 2020 EAF guiding principles of management for achondroplasia

Item	Guiding principle	Vote (%)	Level of agreement (mean; range)
A	Achondroplasia is a lifelong condition requiring lifelong management by an experienced MDT, led by physicians/clinicians experienced in achondroplasia management. Close monitoring during the first two years of life is critical	92	8.9 (8–10)
B	When a diagnosis of achondroplasia is made or suspected, either *in utero* or after birth, the family should be referred as soon as possible to a physician experienced in achondroplasia to discuss the prognosis and management of the condition	100	9.3 (8–10)
C	Decisions around management should be made in the MDT setting jointly with the person with achondroplasia and/or their family	100	9.6 (7–10)
D	The primary goals of management are to enable anticipation, identification and treatment of problems, provide education and support to encourage a healthy lifestyle, positive self-esteem and mental health, autonomy and independence	100	9.2 (8–10)
E	Patients should have access to a variety of adaptive measures, support to ensure proper usage and access to approved treatment options as they become available	91	8.5 (5–10)
F	Regular monitoring in adolescence and adulthood should continue under an MDT with expertise in achondroplasia management. Care should include genetic counselling, transition to adulthood, psychosexual well-being and management of pregnancy	100	9.3 (8–10)

## Methods

Six centres specialist in the management of achondroplasia took part in an exploratory audit to establish the timepoint at which achondroplasia is diagnosed and when families are referred to a specialist centre. Contributors were asked to provide the timepoint of confirmed or suspected diagnosis and the timepoint of referral for their last consecutive 5–10 cases of achondroplasia with unaffected parents. To ensure anonymity the date of birth was identified as Day 0. Where outliers existed or wide variety between countries was observed, the contributing clinician provided further explanation.

The results of the audit were presented and discussed by a group of senior clinicians and surgeons experienced in the diagnosis and management of achondroplasia. The group included paediatric endocrinologists, clinical geneticists, orthopaedic surgeons, a neuropaediatrician, two GPs specialised in achondroplasia, a radiologist, a patient advocate, a genetic counsellor, a neonatologist, an obstetrician, a paediatrician, a paediatric neurosurgeon, and a reference centre coordinator. There was representation from Belgium, France, Germany, Italy, Kingdom of Saudi Arabia, Norway, Portugal, Qatar, Spain, and the UK.

The group was gathered to review the data, identify educational needs, propose strategies to address areas of concern and to make recommendations for best practice.

## Results

Data was collected from six countries, with the timepoint of diagnosis available in 39 cases and timepoint of referral in 45 (six cases from Germany had referral data only). The following centres contributed to the audit: Department of Medical Genetics, Antwerp, Belgium; Department of Genetics, Hôpital Necker-Enfants Malades, Paris, France; Medical Genetics Section, Virgen de la Arrixaca University Hospital, Murcia, Spain; Guy’s and St Thomas’, London, UK; Istituto Gaslini, Genoa, Italy; Universitätskinderklinik, Otto-von-Guericke Universität, Magdeburg, Germany.

**Fig. 1:**
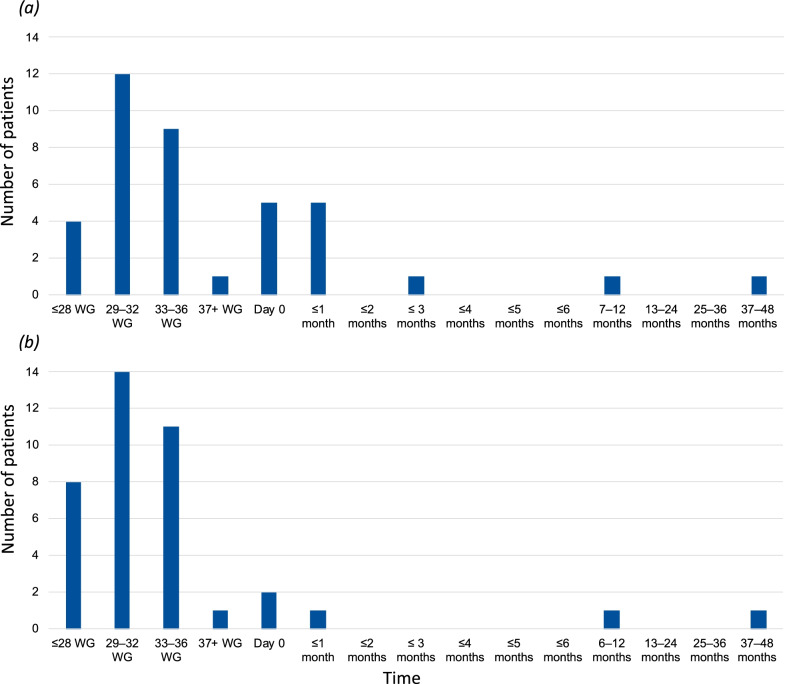
Timepoint of **a** confirmed diagnosis **b** suspicion or confirmation of diagnosis (WG, weeks gestation)

Data from the audit showed that diagnosis is predominantly confirmed prenatally (66.6%), at Day 0 (12.8%) or within one month after birth (12.8%), with centres in France, UK, Italy, and Spain all confirming the diagnosis prenatally or at Day 0 (Fig. 1a). There were two outliers from Belgium. One boy was born in Croatia, had a normal birth length and was suspected to have hypochondroplasia. He tested negative for hypochondroplasia (absence of the p.N540K mutation in *FGFR3*) but no further investigations were carried out until the parents moved to Belgium and presented in Antwerp with their four-year-old son, where the diagnosis of achondroplasia was made. The other patient was a boy who was diagnosed with achondroplasia aged nine months. The relatively inconspicuous clinical characteristics, such as an apparently normal birth length and mild facial features precluded an earlier diagnostic suspicion.

When suspicion of achondroplasia was included in the data, there was a greater proportion of cases identified earlier in the prenatal period (87.1%) and fewer diagnoses at Day 0 (5.1%) or within the first month of life (2.6%) (Fig. 1b).

**Fig. 2:**
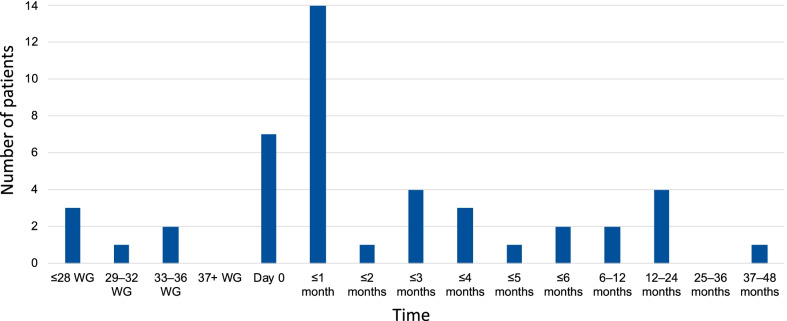
Timepoint of referral to specialist centre (WG, weeks gestation)

There was a wide variety in the timepoint at which cases of achondroplasia are referred to a specialist centre, with the majority of referrals taking place after birth (86.6%), predominantly within the first month (Fig. 2). There was a large difference in the timepoint of referral between countries, with France referring all cases prenatally; Spain between the prenatal period and up to five months; the UK within one month of birth; Italy from Day 0 to two years; Germany up to and including three months to two years; and Belgium from within one month up to and including three months (excluding the outliers previously mentioned).

**Fig. 3:**
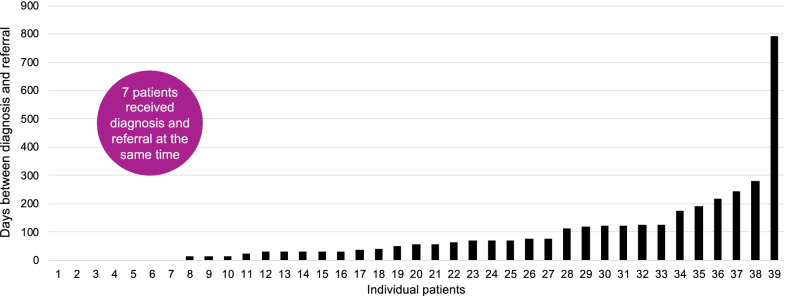
Delay between diagnosis and referral (seven cases received diagnosis and referral at the same time).

A delay between diagnosis and referral was evident. Figure 3 shows the time lapsed between either suspicion or confirmed diagnosis of achondroplasia and referral. Seven patients received diagnosis and referral at the same time (18%). These cases were from France (three), Spain (one) and Belgium (three), although these included the cases that presented at nine months and four years. The time lapse between suspicion or confirmed diagnosis and referral to a specialist centre varied widely between, and even within, centres. In one case from Italy, an infant diagnosed at birth did not present to the Istituto Giannina Gaslini until the parents had concerns for the child aged two years; the time lapsed was 26 months.

## Discussion

It is clear from this audit that while there is some variation in the timepoint of diagnosis of achondroplasia, the majority of cases are suspected or confirmed prenatally, and there is often a delay in referral to a specialist centre. The timepoint of diagnosis achieved in the audit was considered to be adequate, predominantly prenatally or within one month of birth. This supports data from the European population-based epidemiology study by Coi et al. in which the time of diagnosis was prenatal in 63.4% of cases and after birth in 36.6%, of which 24.1% were diagnosed within the first week [1]. The audit demonstrated that the antenatal diagnostic pathway was variable and that a clear structure for diagnosing and referring cases of achondroplasia is needed to improve the prenatal diagnostic rate. The two outliers from Belgium may indicate that in cases of a recorded normal birth length and mild (craniofacial) features the disorder may be missed soon after birth or within the first months.

Molecular confirmation of achondroplasia may not be essential in all situations [5], for example, prenatally if there is no question of termination of pregnancy and an ultrasound diagnosis has been made by an expert in prenatal ultrasonography, in circumstances where invasive testing risks a miscarriage, or when parents do not insist on having a precise diagnosis prenatally and are willing to wait until after the delivery. It may be useful in cases of diagnostic uncertainty [5], however, if an experienced ultrasonographer confidently identifies all indicators of achondroplasia, there may be no need for further imaging or *FGFR3* gene testing. These combined assessments may be sufficient to confirm a diagnosis. These variables may also explain the difference in the timepoints of confirmed diagnosis between the centres in the audit.

The presence of cell-free fetal DNA in maternal blood was first described by Lo et al. [37], and by 2000 Saito et al. had successfully diagnosed achondroplasia using the non-invasive prenatal testing (NIPT) technique [38]. To establish the availability and use of NIPT in Europe, a poll was undertaken at the EAF meeting which showed that this diagnostic technique was available to 13 of 20 attendees. In the UK and France, this technique is validated for *FGFR3*; in Italy the facilities are not routinely available; in Belgium, Spain and Portugal NIPT is not routinely used for single gene mutations. No consensus was reached as to whether NIPT should be recommended as part of best practice.

A similar poll was held for prenatal (invasive) molecular diagnosis. In the majority of countries represented, prenatal molecular diagnosis is common practice (15/18). There is some variety in the type of molecular test used to diagnose achondroplasia. In a non-expert setting the amount of information in a prenatal panel may be overwhelming, and variants of unknown significance can be confusing. Representatives from Spain, Belgium and the UK stated that a targeted skeletal dysplasia panel is used, which limits incidental findings from a full exome analysis, while those from France and Portugal preferred targeted variant analysis, with the option to subsequently carry out a skeletal dysplasia panel if the recurrent achondroplasia *FGFR3* variant is not identified. Single variant targeted testing may be investigated more quickly at laboratories than a panel, enabling a quicker turnaround time; this may be relevant in the prenatal setting when termination of pregnancy is being considered by the parents. The group agreed that for clinicians in a specialist centre who are experienced in achondroplasia, single gene variant sequencing is the most effective way of confirming diagnosis. This supports evidence in the literature that a high index of suspicion for a condition is required to enable targeted molecular testing [21]. If a positive molecular test is achieved prenatally, no further test is required after birth.

In cases where no diagnosis has been made prenatally, the EAF recommend postnatal clinical and radiological evaluation followed by genetic testing to confirm diagnosis of achondroplasia.

### The EAF diagnostic pathway for achondroplasia

**Fig. 4:**
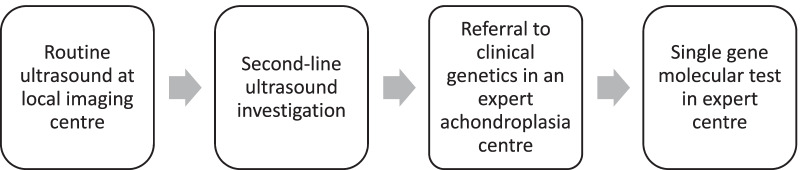
EAF diagnostic pathway for achondroplasia

The EAF agreed that there is a preferred pathway for reaching a confirmed diagnosis of achondroplasia prenatally (Fig. 4). A local imaging centre should provide routine ultrasound screening. If any concerns are raised, referral to the next level of ultrasound investigation, such as a fetal medicine unit, should be made; systemic genetic testing of *FGFR3* upon identification of an isolated shortened femur is not recommended. If there is any indication of a skeletal dysplasia, the family should be referred to a centre with a MDT specialised in the diagnosis and management of skeletal dysplasia. Antenatal findings of achondroplasia are quite specific in the hands of an experienced ultrasonographer, in which case, a targeted gene test can be undertaken to confirm diagnosis.

### The process after diagnosis

It is not always clear where the experts or expert centres in each country are located, and there is no clear national or European-level referral pathway following a diagnosis of achondroplasia. The EAF acknowledge that there may be wide variation in who identifies and manages achondroplasia, so the referral pathway will differ from country to country. Many patient advocacy groups and expert centres have proactive and collaborative channels of communication, which may improve and accelerate access to specialised care. Patient advocacy groups can also facilitate referral in countries where the referral pathway is not clear or where the specialists in achondroplasia are not easily identifiable. Establishing a directory of centres managing achondroplasia and undertaking regular audits of time to diagnosis and referral may assist in identifying the centres managing achondroplasia effectively and support early referral. The EURORDIS 2007 rare disease survey highlights the importance of establishing Centres of Excellence [20]; the creation or recognition of official national or regional reference centres or centres of excellence dedicated to achondroplasia, or to skeletal dysplasia conditions, should be a priority in countries where this is not already established. Close collaboration between centres at a European level may also be beneficial, this may include integration of centres within the European Reference Network on Rare Bone Disorders (ERN-BOND).

Following referral to a specialist centre, the patient journey can vary between countries and between centres. There is no agreed patient journey for individuals with achondroplasia in Europe, or standardised information that is provided. Information on management of achondroplasia can be daunting for parents, and the first contact with several members of the MDT may be overwhelming. Providing the right information at the right time is key, either through the centre or via the patient advocacy group. Direct contact with the family, such as a phone call immediately after referral can prepare them for their first visit to the centre. Genetic counselling and psychological support should also be made available to families on receiving a diagnosis of achondroplasia. Each centre should have clear information available on the MDT organization and the role of each professional. The EAF recommends that best practice is to provide a clear point of contact at the specialist centre, and to clearly explain the MDT structure to families. The EAF plans to develop standard patient journey information that can be adapted to individual centres to support patients on referral.

The EAF guiding principles of management state that *when a diagnosis of achondroplasia is made or suspected, either in utero or after birth, the family should be referred as soon as possible to a physician experienced in achondroplasia to discuss the prognosis and management of the condition*. [8] The results of the exploratory audit identify that immediate referral is not occurring in the majority of cases, with 7/39 patients (18%) receiving diagnosis and referral at the same time. The delay in referral to a specialist centre could be attributed to a number of factors. In many cases of achondroplasia suspected prenatally the parents are informed of the suspected diagnosis and are provided with key information at the time of diagnosis, but may wish to only attend the specialist centre after the child is born, or later if they have no immediate concerns. There are no interventions for the unborn child if achondroplasia is diagnosed *in utero*, so interaction with a specialist centre may be seen as unnecessary until after birth. Families may choose to deliver the child in a hospital close to them, rather than in a specialist centre. There may be processes in individual centres that require investigations and internal referrals prior to referral to an expert centre. In addition, there may be personal and cultural factors involved in the timepoint of diagnosis and referral, such as parents who do not want invasive prenatal diagnosis and are willing to wait until birth for diagnosis. In countries where termination is not an option late in the pregnancy it may not be considered urgent to refer to an expert centre prior to birth; parents may seek centres in other countries where termination is still possible at this time. It should also be acknowledged that some families do not want any further information about achondroplasia on receiving a diagnosis. Some parents may experience a grief response, with initial shock and denial, on receiving a diagnosis of achondroplasia [36, 39]; this may contribute to a delay in referral to a specialist centre. Alternatively, if the child is well, parents may not seek a referral. Many patient advocacy groups share information with parents upon diagnosis and according to their needs at that specific time. The EAF acknowledge all these reasons for a delay in referral, however they advocate for referral as soon as possible after diagnosis to maximise clinical and psychological outcomes. [8]

The length of delay between diagnosis of achondroplasia and referral to a specialist centre warrants further research. Annual auditing of management pathways at individual centres would enable barriers to diagnosis and referral to be identified and strategies put in place to address the challenges. This may include the identification of further educational support, for example, for ultrasonographers, obstetricians and gynaecologists, and the implementation of training to facilitate early diagnosis and timely onward referral on suspicion of achondroplasia.

There are limitations to the EAF Steering Committee audit. The cohort was small (six centres participated), only centres expert in the management of achondroplasia were consulted, data was not captured at different periods of time to highlight differences in the availability of molecular testing, and the wording of the questions could be misconstrued. A repeat of the audit in a larger cohort addressing the limitations would be beneficial to better understand the current situation in Europe, especially in centres lacking expertise in achondroplasia management.

## Conclusions

The complications of achondroplasia in infancy can be life-threatening, with considerations from the delivery of the child, through correct positioning and handling of a newborn, to close monitoring for cervicomedullary compression, among others. The management of complications in infancy and early childhood may impact an individual later in life. Timely and accurate diagnosis is therefore vital, as is timely referral to enable an experienced MDT to fully support individuals with achondroplasia and their families, and to plan appropriate management. The timepoint of diagnosis in the audited centres was not a major concern, however, timely referral to a specialist centre appears to be a greater issue. Collaboration between specialist centres and patient advocacy groups can facilitate communication with parents and individuals with achondroplasia and may improve access to care.

Following the EAF Steering Committee audit, discussion with clinicians who are expert in the diagnosis and management of achondroplasia and a review of the literature, the EAF recommends that:

- Diagnosis of achondroplasia be achieved as early as possible
- If concerns are raised at routine ultrasound, second line investigation should be carried out
- On indication of a skeletal dysplasia, the family should be referred to a centre specialised in the diagnosis and management of achondroplasia
- Genetic testing should be carried out to avoid investigating other skeletal dysplasia conditions
- If a positive molecular test is achieved prenatally, no further test is required after birth
- Clinical and radiological examination supported by molecular testing is the most effective way to confirm diagnosis of achondroplasia after birth
- Individuals and families should be referred to a clinician or MDT specialised in achondroplasia management as soon as possible on suspicion or confirmation of diagnosis
- Each specialist centre should provide a clear point of contact, and should have public information on the MDT organization and the role of each professional
- In countries or regions where there are no official skeletal dysplasia reference or specialist centres, priority should be given to their creation or recognition, together with incentives to improve the structure of the existing MDT managing achondroplasia

The EAF encourage all centres to consider undertaking a similar audit to identify the timepoint of diagnosis and referral, develop strategies to address any delays and improve the process of diagnosis and referral to a centre specialised in the management of achondroplasia.

## Data Availability

Not applicable

## Abbreviations

CT: computed tomography
EAF: European Achondroplasia Forum
ERN-BOND: European Reference Network on Rare Bone Disorders
MDT: multidisciplinary team
NIPT: non-invasive prenatal testing
